# Barriers to and facilitators for the creation, dissemination, implementation, monitoring, and evaluation of oral health policies in the WHO Africa region: A scoping review protocol

**DOI:** 10.12688/f1000research.139689.1

**Published:** 2023-09-15

**Authors:** Alonso Carrasco-Labra, Francisca Verdugo-Paiva, Cleopatra N. Matanhire-Zihanzu, Emmett Booth, Iliana V. Kohler, Olivia Urquhart, Yuka Makino, Michael Glick

**Affiliations:** 1 Center for Integrative Global Oral Health, University of Pennsylvania, Philadelphia, Pennsylvania, 19104, USA; 2 Department of Paediatrics, Obstetrics & Gynaecology and Preventive Medicine and Public Health, Universitat Autonoma de Barcelona, Barcelona, Catalonia, Spain; 3 Epistemonikos Foundation, Santiago, Santiago Metropolitan Region, Chile; 4 Department of Oral Health, Faculty of Medicine and Health Sciences, University of Zimbabwe, Avondale, Harare, P.O Box A178, Zimbabwe; 5 Temple University Libraries, Temple University, Philadelphia, Pennsylvania, USA; 6 Population Studies Center, University of Pennsylvania, Philadelphia, Pennsylvania, 19104, USA; 7 Noncommunicable Diseases Management Team, WHO Regional Office for Africa, Cité Djoué, Brazzaville, P.O. Box 06, Congo

**Keywords:** Scoping review, WHO Africa region, Oral Health Policy, Barriers and Facilitators.

## Abstract

**Background:** Evidence-informed oral health policies (OHP) can be instrumental in ending the neglect of oral health globally. When appropriately developed and implemented, OHP can improve the efficiency of healthcare systems and the quality of health outcomes. However, more than half of the countries in the World Health Organization (WHO) African region did not have an oral health policy or even the existence of a policy in need of additional and more national-specific OHP as part of non-communicable diseases and universal health coverage agendas. The objective of this protocol’s study is to determine the barriers to and facilitators for the creation, dissemination, implementation, monitoring, and evaluation of OHP in the WHO Africa region.

**Methods:** We will conduct a systematic search in Global Health, Embase, PubMed, PAIS, ABI/Inform, Web of Science, Academic Search Complete, Scopus, databases that index gray literature, and the WHO policy repositories. We will include qualitative, quantitative, or mixed-methods research studies and OHP documents published since January 1, 2002, which address stakeholders’ perceptions and experiences regarding barriers to and facilitators for the creation, dissemination, implementation, monitoring, and evaluation of OHP in countries part of the WHO African region. We will produce descriptive statistics (frequencies and proportions) for quantitative data and conduct descriptive content analysis for qualitative data.

**Discussion**: To effectively establish evidence-based OHP in the WHO African region, it is crucial to recognize existing challenges and opportunities for progress. The findings of this review will be relevant for Chief Dental Officers at ministries of health, administrators of dental schools, or academic institutions in the WHO African region and will inform a stakeholder dialogue meeting in Kenya in November of 2023.

**Registration:** Open Science Framework: https://doi.org/10.17605/OSF.IO/9KMWR

## Introduction

Oral health is a topical health discipline neglected in global, regional, and national health matters. ^1^ Oral diseases are among the most common non-communicable diseases (NCDs) in the African Region. In 2016, oral diseases affected approximately 45% of the population in the World Health Organization (WHO) African Region. ^2^ In the previous 30 years, among all WHO regions, the African region has the largest increase in oral disease burden. ^1^ In 2019, more than 70% of countries in the WHO African region spent less than USD 1 per person per year on oral health care. The region also has a long-term problem of a limited oral health workforce. ^1^

Evidence-informed policies can be instrumental in ending the neglect of oral health globally, ^3^ since policies, when properly developed and implemented, can have the ability to improve the efficiency of healthcare systems and the quality of health outcomes. ^4^ In 1998, the WHO African Region Committee proposed a strategy to strengthen countries’ capacity to improve community oral health by effectively using proven interventions to address oral health needs. At that time, only 14 countries in the WHO African Region (30%) had national oral health policies (OHP). ^5^ Currently, several countries in the WHO African Region lack the necessary national OHP. By 2020, two decades later, only 19 countries confirmed having a national health policy plan when conducting the mid-term assessment of the latest regional strategy on oral health 2016-2025 to address oral diseases as part of NCDs and universal health coverage (UHC) agenda. ^6^ The absence of evidence-informed OHP is a critical concern contributing to an increased oral disease burden, oral health workforce shortage, and inadequate oral health service provision. ^3^

The FDI Vision 2030 Report, ^7^ the Lancet Commission in Oral Health, ^3^ the landmark 2021 WHO Oral Health Resolution, ^8^ along with the WHO Global Strategy for Oral Health ^9^ and its action plan have been highlighting an urgent need for immediate health systems enhancements, aiming to achieve UHC for oral health. The 2022 Global Oral Health Status Report notes that there is an pressing need to address the high oral disease burden and that policy-makers and various stakeholders will be instrumental in prioritizing oral health advocacy at a global, regional, and national level as part of NCDs and UHC agenda. ^1^ To ensure the adoption and implementation of these frameworks in the African region, national OHP should be developed and aligned with global and regional strategies.

To accelerate the optimal creation, dissemination, implementation, monitoring, and evaluation of OHP in the WHO African region, understanding the current state of existing policies, including barriers and facilitators, is warranted. We present a protocol for a scoping review to determine what are the barriers to and facilitators for the creation, dissemination, implementation, monitoring, and evaluation of oral health policies in the WHO Africa region.

## Protocol

We used the Preferred Reporting Items for Systematic Reviews and Meta-Analysis Protocols (PRISMA-P) ^10^ and we will use the PRISMA-ScR extension for scoping reviews to present our findings. ^11^

### Definitions

*Oral health definitions*

An article addressing oral health should cover aspects included in the following definitions:

1. “ *Oral health is multi-faceted and includes the ability to speak, smile, smell, taste, touch, chew, swallow and convey a range of emotions through facial expressions with confidence and without pain, discomfort and disease of the craniofacial complex (head, face, and oral cavity).*” ^12^
2. “ *Oral health is the state of the mouth, teeth and orofacial structures that enables individuals to perform essential functions, such as eating, breathing and speaking, and encompasses psychosocial dimensions, such as self-confidence, well-being and the ability to socialize and work without pain, discomfort and embarrassment.*” ^9^

As an extension, articles focusing on modifiable risk factors for oral diseases such as sugar consumption, tobacco use, alcohol use, and oral hygiene, and their underlying social and commercial determinants will also be included.

*Health policy definition*

We will consider a health policy any statement made by an organization, or a group of organizations, at the national or regional level (regions of the continent of Africa, e.g., Sub-Saharan Africa) that recommends or suggests a particular course of action or options related to health and directed to patients, caregivers, healthcare professionals, institutions, or organizations. In this review, a health policy may include a strategy (a long-term plan designed to achieve a particular goal) or an action plan (a scheme of course of action, which may correspond to a policy or strategy, with defined activities indicating who does what, when, how and with what resources to accomplish an objective). ^13^

### Eligibility criteria

**Inclusion criteria**

We will include articles providing quantitative evidence or qualitative statements about barriers to and facilitators for creating, disseminating, implementing, monitoring, and evaluating OHP in the WHO African region.

*Population*

We will include studies that focus on characterizing the perceptions and experiences of a variety of stakeholder groups, including policy-makers, healthcare managers, administrators, organizational leaders (including non-governmental organization leaders), healthcare professionals, researchers, and citizens. We will use the definition of citizens proposed by the Evidence Commission report, which includes all members of society (e.g., patients and caregivers, service users, parents, voters, community leaders, and workers). ^14^

*Setting*

We will include studies conducted in any of the 47 countries of the WHO African Region: Algeria, Angola, Benin, Botswana, Burkina Faso, Burundi, Cabo Verde, Cameroon, Central African Republic, Chad, Comoros, Congo, Cote d’Ivoire, Democratic Republic of Congo, Equatorial Guinea, Eritrea, Eswatini, Ethiopia, Gabon, Gambia, Ghana, Guinea, Guinea Bissau, Kenya, Lesotho, Liberia, Madagascar, Malawi, Mali, Mauritania, Mauritius, Mozambique, Namibia, Niger, Nigeria, Rwanda, Sao Tome and Principe, Senegal, Seychelles, Sierra Leone, South Africa, South Sudan, Togo, Uganda, United Republic of Tanzania, Zambia, and Zimbabwe. ^15^

*Type of evidence*

We will include articles published since January 1, 2002, in English, French, or Portuguese, and falling between two types of publications addressing barriers to and facilitators for OHP:

1. Research articles (systematic reviews, scoping reviews, umbrella reviews, primary studies, and conference proceedings):
  • Research articles that directly assess or measure barriers to and facilitators for OHP creation, dissemination, implementation, monitoring, and evaluation. The assessment of barriers and facilitators could have been done in a single dental clinic in the WHO Africa region at a national, regional (e.g., a province or territory), or community level (e.g., a village, a municipality).
  • Research articles exploring perceptions, experiences, perspectives, knowledge, or attitudes regarding oral health care services (financing, access, or provision) for strengthening healthcare systems in the WHO African region. The article can cover barriers or facilitators across several policies.
  • Research articles exploring perceptions, experiences, perspectives, knowledge, or attitudes related to specific interventions to diagnose, treat, or prevent oral diseases in the WHO African region. These studies will be included if the intervention is connected to an existing oral health policy or government program (the research article should report or reference in the introduction, methods, or discussion evidence of a connection between the study purpose and specific oral health policies or programs).
2. Oral health policy-related documents (national policies, clinical practice guidelines, national strategies and plans, documents written by authors that are part of Ministries of Health or other policy-oriented governmental global and local organizations, policy briefs, and policy analysis):
  • Health policy documents are eligible if the document has a section or a sentence reporting barriers to or facilitators for oral health policy creation, dissemination, implementation, monitoring, and evaluation.

**Exclusion criteria**

We will exclude research articles and oral health policy-related documents meeting one of the following criteria:

• Conference abstracts, commentaries, editorials, and protocols.
• Articles assessing prevalence, burden of disease, or epidemiological data from oral diseases.
• Articles assessing risk or protective factors for oral diseases.
• Articles evaluating the effectiveness or safety of interventions to prevent, diagnose, or treat oral diseases.
• Articles reporting economic evaluations of interventions to prevent, diagnose, or treat oral diseases.
• Articles reporting only aggregated data on barriers and facilitators, including a combination of countries within and outside the WHO African region.

### Outcomes

We will extract quantitative data and qualitative statements informing the existence of barriers and facilitators. We define a barrier as any practical, political, financial, or technical problem preventing or obstructing any OHP aspect. A facilitator corresponds to an entity, process, technology, legislation, or organizational structure that promotes or optimizes any aspect of an OHP.

We will classify barriers and facilitators according to the specific aspect of an OHP that is affected. First, an article classified as addressing issues of OHP creation should describe the development of a health policy for individuals, patients, caregivers, healthcare professionals, institutions, or organizations (e.g., studies or documents assessing stakeholder engagement in health policy development, use of research evidence in policy creation, researchers, policymakers, and other stakeholders’ involvement in health policy dialogue). These articles can also describe the adaptation or adoption of health policies, guidelines, or guidance developed elsewhere for local use, that is, the use of existing documents produced in one setting to be modified for use in another setting (e.g., studies reporting African guideline developers adopting existing recommendations from other countries). ^16^ Articles describing plans and strategies to share, distribute and promote health policy content and ensure the audiences of interest are reached (e.g., translation into multiple languages, open-access publications, engagement of intermediaries, mass media campaign, email distribution) will be classified as describing issues of OHP dissemination. Articles relating interventions to apply the health policy content in the healthcare system, monitor policy implementation, evaluate its impacts or changes in healthcare organizations, the behavior of healthcare professionals, patients, caregivers, or the use of health services by healthcare recipients (e.g., printed educational materials, audit, and feedback, reminders, financial incentives, computer decision support systems) will be classified as addressing issues of implementation, monitoring, and evaluation. Second, we will also classify the barriers and facilitators based on the WHO building blocks pillars for health systems. ^17^

### Electronic searches

We will search the following databases: Global Health, Embase, PubMed, Public Affairs Information Service Index (PAIS), ABI/Inform, Web of Science, Academic Search Complete, and Scopus. In addition, databases that index gray literature (Dissertations Global, Google Scholar) will be searched. To ensure the retrieval of relevant materials produced by WHO, additional sources of information will be searched (WHO’s Institutional Repository for Information Sharing (IRIS), Google, WHO Noncommunicable Diseases Document Repository, and the WHO’s African members’ oral health policies repository). Materials indexed in African regional databases such as Regional African Index Medicus and African Journals Online are covered in the databases mentioned above.

The specific search strategies for each database will be created by two information specialists with expertise in systematic review searching and health and social sciences. The search strategies will be developed by the specialists with input from the project team. The search strategies will be peer-reviewed by other information specialists not otherwise associated with the project. A link to the draft PAIS search strategy can be found in the extended data section. ^24^ After the PAIS search strategy is finalized, it will be adapted to the syntax and thesauri of the other databases.

### Selection of studies

Pairs of reviewers will independently screen titles and abstracts against the eligibility criteria. Retrieved documents published in French or Portuguese will be designated to a research team member who comprehends each of these languages. We will obtain full reports for all records that appear to meet the eligibility criteria or require further analysis to decide their inclusion. A third review author will resolve any discrepancies. We will use Covidence ^18^ to manage the screening process, upload search results, screen titles abstracts, and full-text articles, and resolve disagreements. We will record the reasons for citation exclusion at the full-text level.

### Data extraction and management

Pairs of reviewers will independently extract data from each included article using a data extraction sheet specifically designed for this review. We will collect the following information: first author, publication year, type of article, country, setting, group or audience to which the policy was directed, participants’ characteristics, study objectives, study research methods, and key findings. We will also extract all relevant evidence regarding stakeholders’ perceptions and experiences regarding barriers and facilitators for the creation, dissemination, implementation, monitoring, and implementation of OHP. This includes qualitative, quantitative, or mixed data from participants’ quotes, narrative descriptive summaries, author hypotheses, theoretical frameworks, explanations and recommendations, themes, and sub-themes. The draft data extraction form will be modified and adapted as necessary during the extraction process. Modifications will be detailed as part of the methods of our review. We will discuss disagreements, and one arbiter will adjudicate unresolved discrepancies.

### Analysis and data synthesis

We will use descriptive statistics, including frequencies and proportions when analyzing quantitative data. Qualitative data will be analyzed using descriptive content analysis. ^19^ Since this is the first review of its kind in oral health, we will combine two information sources to create a new taxonomy to classify identified barriers and facilitators. First, we will use taxonomies from previous systematic reviews addressing barriers and facilitators for health policies outside the scope of oral health. ^20^ Second, in an iterative process, we will use statements identified from the included articles reflecting barriers and facilitators specific to oral health, and revise our taxonomy.

After establishing an initial taxonomy, we will categorize the extracted data across the different categories. Two researchers will independently review and apply the coding to the included studies’ data and documents. The team will discuss discrepancies in coding to achieve consensus. Factors that affect OHP will be coded as barriers or facilitators against a predefined list of factors that will be iteratively updated and defined as new factors are identified. A summary of the data coded to barriers and facilitators will be presented in tables.

## Discussion

To make the needed transformations to ensure rigorous evidence-based OHP in the WHO African region, it is essential to understand the current challenges and opportunities for their creation and implementation. Hence, this scoping review aims to identify and summarize the available research evidence and stakeholders’ perspective and experiences regarding the barriers and facilitators for OHP. This review will help to appreciate contextual factors, actors, stakeholders, policy processes, and other dynamics influencing the entire policy enterprise. ^21^ In addition, it will help to identify challenges that have limited oral health system performance, particularly in low-resource settings such as the African context. ^22^ Our findings can also assist in designing cost-effective initiatives to overcome the barriers, optimizing resource utilization, and enabling the effective adoption and implementation of the WHO Global Oral Health Strategy. ^23^

Some limitations of this review relate to the complexity in nature, design, and availability of articles to be retrieved. For example, preliminary piloting of potentially eligible articles proved that barriers and facilitators are widely reported, often not explicitly labeled as such, and rarely supported with references or data. The broad scope of our work warrants the application of a variety of quantitative and qualitative synthesis techniques. In addition, we expect that some eligible references may not be available in full text, even for members of the review team based in Africa. Our review’s strength relies on the comprehensive and multidisciplinary creation of a search strategy covering global and regional databases and considering policy-specific sources for Africa. Another strength is the collaboration with the Noncommunicable Diseases Management Team at the WHO Regional Office for Africa, whose input ensures that the review findings are relevant for stakeholders and other users.

### Dissemination

The findings of this scoping review will be disseminated using a combination of strategies:

publications in peer-review journals, abstract presentations in research and oral health policy related-conferences, circulation of executive summaries and policy briefs with Chief Dental Officers at ministries of health, deans of dental schools or academic institutions in WHO African region countries, and the organization of a stakeholder dialogue meeting in Kenya (November 2023), which this work will partially inform.

### Study status

Creation of the search strategy has been completed. Data collection has begun and analysis is expected to be completed by October 2023.

#### Ethical considerations

As there are no human participants involved, there will be no requirement for ethical approval. Patients and/or the public were not involved in the design of this protocol.

## Data Availability

No underlying data are associated with this article. figshare: Search strategy. Barriers to and facilitators for the creation, dissemination, implementation, monitoring, and evaluation of oral health policies in the WHO Africa region, https://doi.org/10.6084/m9.figshare.23791923. ^24^ Data are available under the terms of the Creative Commons Attribution 4.0 International license (CC-BY 4.0).
