# Supplementary material for: Barriers to and facilitators for the creation, dissemination, implementation, monitoring, and evaluation of oral health policies in the WHO Africa region: A scoping review protocol
Source: F1000Res. 2024 Mar 15;12:1160. Originally published 2023 Sep 15. [Version 2] doi: 10.12688/f1000research.139689.2 (PMC10988199; doi:10.12688/f1000research.139689.2)
Supplement: Peer review history

# Reviewer report: Reviewer response for version 2

**DOI:** 10.5256/f1000research.163680.r256362

**Published:** 2024-04-02

**Authors:** Mpho Molete

**Affiliations:** 1 Department of Oral Biological Sciences, School of Oral Health Sciences, Faculty of Health Sciences, University of the Witwatersrand Johannesburg, Johannesburg, Gauteng, South Africa

The study protocol has improved, and the authors have now provided the clarity that was needed.

 Please remove "descriptive" behind the word content analysis. The analysis is just: Content analysis.

 I am satisfied with the reviewed protocol.

Is the study design appropriate for the research question?

Yes

Is the rationale for, and objectives of, the study clearly described?

Yes

Are sufficient details of the methods provided to allow replication by others?

Partly

Are the datasets clearly presented in a useable and accessible format?

Not applicable

Reviewer Expertise:

Oral health epidemiology, Child Oral Health, Implementation Science

I confirm that I have read this submission and believe that I have an appropriate level of expertise to confirm that it is of an acceptable scientific standard.

# Reviewer report: Reviewer response for version 1

**DOI:** 10.5256/f1000research.152986.r231587

**Published:** 2024-02-21

**Authors:** Mpho Molete

**Affiliations:** 1 Department of Oral Biological Sciences, School of Oral Health Sciences, Faculty of Health Sciences, University of the Witwatersrand Johannesburg, Johannesburg, Gauteng, South Africa

This is an important relevant enquiry as oral health in the African continent is neglected, not much funding goes into addressing its challenges, and yet 45% of the population living in the WHO African Region are affected by oral diseases. Therefore, effective policy processes are necessary for improving oral healthcare systems and outcomes around the continent.

 The proposal thus aims to undertake a scoping review that includes peer reviewed articles and grey literature as well. This is hoped to assist in addressing the gaps in terms of integrating oral health with NCD's and giving direction for Universal Health Coverage.

 The significance of the review is well highlighted, however there is concern that the objectives are too broad and may hinder the authors from obtaining rich insightful information. For instance, very little monitoring and evaluation information may be found as much literature has shown there to be paucity in M&E of not only of oral health policies but interventions across the continent.

 In terms of methodology, the inclusion criteria needs to be refined as it is ambiguous. There needs to be clarity on how each criterion is different from the other or else screening of the articles will be difficult.

 I also suggest that the overarching search strategy with the search terms used must be indicated in the protocol so that we have an idea on what was guiding the information specialist. 

 There needs to be clarity on how many reviewers will be used to analyze papers in French and Portuguese.

 Lastly please check your use of tenses, particularly in the abstract.

Is the study design appropriate for the research question?

Yes

Is the rationale for, and objectives of, the study clearly described?

Yes

Are sufficient details of the methods provided to allow replication by others?

Partly

Are the datasets clearly presented in a useable and accessible format?

Not applicable

Reviewer Expertise:

Oral health epidemiology, Child Oral Health, Implementation Science

I confirm that I have read this submission and believe that I have an appropriate level of expertise to confirm that it is of an acceptable scientific standard, however I have significant reservations, as outlined above.

# Reviewer report: Reviewer response for version 1

**DOI:** 10.5256/f1000research.152986.r207139

**Published:** 2023-10-16

**Authors:** Manu Mathur

**Affiliations:** 1 Queen Mary University of London, London, England, UK

This is a protocol of a very interesting study. These are the water shed years for global oral health and this study will help in putting oral health at the forefront for the debates on strengthening health systems in Africa. There are a few bits and bobs that needs more elaborate work. Notably:

- Introduction Line 2: Needs a reference.
- Introduction: Africa is in a very nascent stage of building evidence on health policy and systems. Do you think that evidence informed policy is even feasible when the research is very sparse? I think it is important that you define what you mean by evidence informed policy making in the Introduction and then move forward.
- Methodology: Why 2002 is taken as a cut off date for inclusion of articles? This needs to be justified.
- Type of evidence: Point Number 2: not entirely clear. Generally policy documents don’t have this information. Does that mean that all health policy documents will be excluded? Need some clarification.
- Data Analysis: What sort of quantitative data are you expecting to obtain? This needs to be elaborated.

Is the study design appropriate for the research question?

Yes

Is the rationale for, and objectives of, the study clearly described?

Yes

Are sufficient details of the methods provided to allow replication by others?

Partly

Are the datasets clearly presented in a useable and accessible format?

Not applicable

Reviewer Expertise:

Public health, oral health, health policy and systems research

I confirm that I have read this submission and believe that I have an appropriate level of expertise to confirm that it is of an acceptable scientific standard.
